# Subjective accelerated aging moderates the association between COVID-19 health worries and peritraumatic distress among older adults

**DOI:** 10.1017/gmh.2021.13

**Published:** 2021-04-14

**Authors:** Lee Greenblatt-Kimron, Lia Ring, Yaakov Hoffman, Amit Shrira, Ehud Bodner, Yuval Palgi

**Affiliations:** 1 School of Social Work, Ariel University, Ariel, Israel; 2 Department of Gerontology, University of Haifa, Haifa, Israel; 3 Interdisciplinary Department of Social Sciences, Bar-Ilan University, Ramat-Gan, Israel

**Keywords:** COVID-19, health worries, peritraumatic distress, subjective accelerated aging

## Abstract

**Background:**

The present study examined whether subjective accelerated aging moderated the relationship between COVID-19 health worries and COVID-19 peritraumatic distress among older adults.

**Method:**

The sample consisted of 277 older adults (M = 69.58, s.d. = 6.73, range 60–92) who answered an online questionnaire during the outbreak of COVID-19 pandemic in Israel. Participants completed the measures of background characteristics, exposure to COVID-19, COVID-19 health worries, subjective accelerated aging and COVID-19-based peritraumatic distress.

**Results:**

Higher levels of COVID-19 health worries were correlated with higher levels of peritraumatic distress symptoms among older adults. Moreover, those reporting accelerated aging also reported a higher level of peritraumatic distress. Finally, the interaction between COVID-19 health worries and subjective accelerated aging predicted peritraumatic distress, suggesting that COVID-19 worries were associated with peritraumatic distress to a stronger degree among older adults who felt they were aging faster.

**Conclusions:**

These findings indicate that negative views of aging may serve as an amplifying factor for traumatic distress during the COVID-19 pandemic. Although preliminary, the findings provide insight for potential screening and interventions of older adults at risk of developing peritraumatic distress symptoms during the global pandemic.

## Introduction

On 11 March 2020, the World Health Organization (WHO) declared that the coronavirus disease 2019 (COVID-19) outbreak could be characterized as an international pandemic as the virus had spread increasingly worldwide (WHO, 2020). COVID-19 is a severe and persistent virus, by 19 March 2021, the morning after the Israeli government announced the fourth step out of the third nationwide lockdown, 826 217 Israeli citizens had been tested positive for COVID-19, with 6071 deaths, most of them older adults (Ministry of Health, Israel, 2021). The aging population in particular has been one of the most vulnerable, with a high number of older adults infected globally (Niu *et al*., 2020). Preliminary data have attested that COVID-19 has adverse psychological outcomes (Qiu *et al*., 2020) such as depressive symptoms, anxiety, and high levels of worries and stress (Wang *et al*., 2020*a*; Palgi *et al*., 2020*b*). Likewise, COVID-19 has resulted in fear (Ahorsu *et al*., 2020*a*, *b*), depression, anxiety (Palgi *et al*., 2020*b*), and death anxiety (Ring *et al*., 2020) among the older population. However, older adults suffering from chronic medical conditions related to increased risk of death due to COVID-19 were not found to be at higher risk for depressive and anxiety symptoms (Palgi *et al*., 2020*b*).

In a systematic review of 43 studies on the mental health consequences of the COVID-19 pandemic, the researchers found a high level of post-traumatic stress symptoms and depressive symptoms among COVID-19 patients (Vindegaard & Benros, 2020). Moreover, patients with preexisting psychiatric disorders experienced a deterioration of symptoms. Studies revealed an increase in psychiatric symptoms such as depression, anxiety, psychological distress, and poor sleep quality among health care workers. Studies also showed lower psychological well-being and higher levels of anxiety and depression in the general public than before the COVID-19 pandemic (Vindegaard & Benros, 2020). Nevertheless, there is minimal evidence available on the mental health implications of prior medical pandemics (Tsang *et al*., 2004; Sood, 2020). Consequently, an urgent plea to the academic world was made to conduct trauma research during the COVID-19 pandemic (Horesh and Brown, 2020). This is particularly important among older adults, which the trauma literature has understudied throughout the years. The present study, therefore, aimed to provide data that would shed light on the role that views of aging play in the traumatic distress experienced among the older population during the COVID-19 pandemic. Specifically, the study aimed to examine subjective accelerated aging as moderating the link between COVID-19 health worries and peritraumatic distress among older adults.

### Peritraumatic distress

Peritraumatic distress relates to cognitive and emotional distress throughout and immediately following a traumatic event (Brunet *et al*., 2001; Palgi *et al*., 2020*a*). A person's peritraumatic encounter is determined by several determinants, such as the continuance of exposure and proximity to the event (Kannis-Dymand *et al*., 2019). Peritraumatic distress symptoms have been positively correlated with posttraumatic stress disorder (PTSD) symptoms (e.g. Thomas *et al*., 2012; Palgi *et al*., 2020*a*) after both human-made (Simeon *et al*., 2003) and natural catastrophes (Basoglu *et al*., 2004; Hollifield *et al*., 2008). Prior examinations of mass events, such as natural catastrophes, typically are accompanied by adverse effects on mental health, in particular with PTSD (Makwana, 2019). It has already been noted that the unexpected outbreak of a disease bears a threat to the mental health of the population, as demonstrated in China during the initial stages of the COVID-19 breakout (Ahmed *et al*., 2020). Therefore, the current pandemic is a high-risk emergency for psychiatric morbidity (Sood, 2020), with a major concern being the development of PTSD (Banerjee, 2020). As mentioned, peritraumatic distress is known to be associated with PTSD (e.g. Palgi *et al*., 2020*a*); thus, we examined peritraumatic distress among older adults, who are considered at higher risk for severe COVID-19 complications (Emami *et al*., 2020) and may, therefore, report COVID-19 worries. We thus sought to explore the relationship between COVID-19 health worries and peritraumatic distress among older adults.

### COVID-19 health worries

Worries refer to fundamental cognitive features of anxiety, characterized by the repeated occurrence of ideas, thoughts and images about possible adverse events, which are relatively uncontrollable (Borkovec *et al*., 1983; Williams, 2013). Worries may impact well-being and mental health if they appear repeatedly, resulting in a harmful level of anxiety (Reis *et al*., 2019). Nevertheless, worries are not confined to patients with anxiety disorders or to a particular emotion, but are rather relationally rooted in distinct settings (Lutz and White, 1986). Worries have been found to be a focus in health-related issues, with numerous studies reporting an association between worries and medical illnesses, such as cancer patients (e.g. Roy *et al*., 2019), patients with heart failure (e.g. Bagheri *et al*., 2018), Crohn's disease patients (Wåhlin *et al*., 2019), and individuals suffering from asthma (e.g. Islamovic *et al*., 2019). During an influenza outbreak, 10–30% of the general public reported being very or moderately worried about the probability of contracting the virus (Rubin *et al*., 2010). In line with this notion, recent studies have indicated that since the outbreak of COVID-19, levels of fears and worries have increased (Lin, 2020). The rise in health worries is understandable, due to the relatively high morbidity risk of COVID-19 (Kobayashi *et al*., 2020; Sood, 2020), the vagueness of the situation, fears about the safety of the new vaccine in Israel, the leading country to operate a COVID-19 vaccine program, regarding the vaccine's long-term effects (Ministry of Health, Israel, 2021), and the need for quarantine for undetermined periods (Ahmed *et al*., 2020).

Since the COVID-19 breakout, constant worrying has already been noted in the population, with differences in severity (Sihag and Kumar, 2020). Moreover, as old age has been determined to be a major risk factor for COVID-19 complications (Emami *et al*., 2020; Wang *et al*., 2020*b*), it has also been proposed that older adults are expected to have higher stress levels during the COVID-19 pandemic (Kar, 2020). The rationale for examining the potential link of accelerated aging with COVID-19 worries is based on several issues. First, higher levels of stress were observed in older adults due to COVID-19 (Kar, 2020). Second, older adults are at higher risk for COVID-19 complications (Santesmasses *et al*., 2020). Third, PTSD which is closely linked with peritraumatic stress has been addressed as a secondary COVID-19 outcome (Dutheil *et al*., 2020). Fourth, the recent findings (Palgi, 2020) showing positive linkage between trauma exposure, challenges of aging, and accelerated aging.

### Subjective accelerated aging

The term subjective age refers to how old people perceive themselves to be (Stephan *et al*., 2015). Studies have reported an association between subjective age and adverse mental and physical outcomes (e.g. Choi and DiNitto, 2014), including traumatic exposure (Schafer, 2009), acute stress disorder (Hoffman *et al*., 2015) and PTSD symptoms (Solomon *et al*., 2009; Hoffman *et al*., 2016). An older subjective age has also been reported as both moderating the PTSD-posttraumatic growth link (Palgi, 2016), as well as strengthening the adverse effect of posttraumatic symptoms on successful aging (Shrira *et al*., 2016).

Based on the subjective weathering hypothesis, which asserts that subjective aging is a key aspect of the stress process (Benson, 2014), it may be assumed that people tend to cognitively divert their internalized clock and form an older age identity due to traumatic events (Hoffman *et al*., 2016; Palgi, 2016). Moreover, a similar psychological process paralleling the biological process of accelerated aging occurs (Palgi, 2020). Accordingly, age identity has been reported not as a defined measure, but rather an identity that may oscillate during difficult life events (Schafer and Shippee, 2010). Based on these theories, Palgi (2016) suggested that coping with trauma exposure, while coping with the challenges of aging, may bring people to feel their aging process is accelerated. This has been demonstrated among ex-prisoners of war with high levels of PTSD symptoms, who demonstrated shortened telomere length – an indicator of neural accelerated aging (Tsur *et al*., 2018) as well as an older subjective age (Avidor *et al*., 2016). Yet, given the abovementioned association between objective and subjective measures of accelerated aging, indicating the awareness people have to their physical aging process, subjective accelerated aging can be examined directly (at a single time point) to assess one's feeling of aging acceleration across time. This assumption is based on previous studies, which examined the effects of subjective age on older adults, and which found subjective age to be a better predictor of psychological and health-related functioning (Kotter-Grühn *et al*., 2009) than chronological age was. Nevertheless, to the best of our knowledge, only two studies to date specifically asked whether participants experienced accelerated aging (Bergman and Palgi, 2020; Palgi, 2020); results reveal that accelerated aging was linked with both higher levels of PTSD symptoms and lower levels of positive mental health with this single item querying subjective accelerated aging in a cross-sectional manner.

In summary, based on the above, the present study aimed to assess if COVID-19 worries are linked with peritraumatic distress symptoms in older adults. Additionally, we asked if subjective accelerated aging would moderate this link. It was first hypothesized that higher levels of COVID-19 health worries would be related to peritraumatic distress among older adults. The second hypothesis maintained that subjective accelerated aging would moderate this association. Namely, relative to lower levels of accelerated aging, for persons with higher levels of accelerated aging, there would be a stronger positive relationship between COVID-19 health worries and peritraumatic distress among older adults.

## Method

### Participants and procedure

The current study used data drawn from an online survey in Israel between 16 March and 14 April 2020. On 14 April 2020, the last day of data collection, 12 046 Israeli citizens were tested positive for COVID-19, with 123 deaths, most of them older adults (https://www.worldometers.info/coronavirus/country/israel/). Participants included 277 older adults between the ages of 60 and 92, with an average age of 69.58 (s.d. = 6.73). The majority of the participants were women (*n* = 191, 69.0%), married/living with a partner (*n* = 204, 73.6%), and had tertiary academic education (*n* = 201, 72.8%). Almost half of the participants self-rated their health as good/very good (*n* = 178, 64.5%). Less than half of the participants reported having chronic diseases which are associated with increased medical complications due to COVID-19 (*n* = 115, 42.9%). The data collection process began after receiving IRB approval from the Ethics Committee at Bar-Ilan University. All participants signed an electronic informed consent form before completing the questionnaire. The background characteristics of the study sample are presented in Table 1.

**Table 1.** Descriptive statistics for the study variables (*N* = 277)

	M/%	s.d.	1	2	3	4	5	6	7	8	9
1. Age	69.58	6.73	–								
2. Gender^a^	69.0%	–	−0.13*	–							
3. Marital status^b^	73.6%	–	−0.06	−0.22***	–						
4. Education	5.56	0.86	−0.12*	0.09	0.06	–					
5. Self-rated health	3.71	0.93	−0.23***	0.20**	−0.02	0.21***	–				
6. Chronic diseases^c^	42.9%		0.25***	−0.23***	−0.03	−0.16*	−0.51***	–			
7. Exposure to COVID-19	1.20	1.05	−0.11	0.01	0.03	−0.00	0.12*	−0.03	–		
8. COVID-19 health worries	2.98	0.90	−0.12	−0.05	0.06	−0.15*	−0.20**	0.01	0.00	–	
9. Subjective accelerated aging	2.31	0.90	0.06	−0.06	0.02	−0.18**	−0.24***	0.13*	0.08	0.15*	–
10. Peritraumatic distress	9.46	6.51	−0.10	0.13*	−0.04	−0.20**	−0.13*	−0.05	−0.01	0.40***	0.38***

a Women.

b Married or living with partner.

c Has chronic diseases.

*p < 0.05, **p < 0.01, ***p < 0.001.

### Measures

*Background characteristics* included age, gender, marital status rated as 1 (*single, widow, divorced*) and 2 (*married or leaving with a partner*), level of education was classified into one of six categories in line with the International Standard Classification of Educational Degrees (ISCED-97) (United Nations Educational, Scientific, and Cultural Organization, 1997) ranging from 1 (*without formal education*) to 6 (*formal tertiary education*) and health status rated from 1 (*not at all good*) to 5 (*very good*). In addition, participants were asked if they suffered from chronic diseases (i.e. cardiovascular disease, diabetes, chronic respiratory disease, hypertension, and cancer), which have been associated with increased medical complications due to COVID-19.

*Exposure to COVID-19* was assessed by respondents answering *yes* or *no* to six questions regarding exposure to the coronavirus (i.e. currently or previously being in quarantine, having had coronavirus, knowing people, family members, or friends in quarantine or who had coronavirus). The exposure score was the sum of events the participant was exposed to.

*COVID-19 health worries* were assessed by a four-item scale designed for this study. Participants rated their levels of health worries about self or close relatives and friends developing COVID-19, or infecting others by COVID-19 (i.e. how worried are you about: being infected by the coronavirus, about people close to you likely to have been infected by the coronavirus, about one of your family members likely to be infected by the coronavirus, about carrying the coronavirus, and about infecting those close to you) on a five-point scale from 1 (*completely disagree*) to 5 (*completely agree*). In this study, Cronbach's *α* for COVID-19 health worries was 0.78.

*Subjective accelerated aging* was assessed by a single item (adapted from Palgi, 2020). Participants rated their subjective accelerated aging on a five-point scale, by answering the question ‘On the whole, I feel that due to the COVID-19: 1 (*my aging rate is very slow*) to 5 (*my aging rate is very fast*)’. Higher scores indicate higher subjective accelerated aging.

*Peritraumatic distress* symptoms were assessed by the 13-item Peritraumatic Distress Inventory (PDI; Brunet *et al*., 2001). Participants rated their symptoms by referring to COVID-19 outbreak on a five-point scale from 0 (*not at all*) to 4 (*extremely true*). The peritraumatic distress score was the sum of ratings, with higher scores indicating greater distress. Cronbach's *α* in a previous study that used the Hebrew translation was 0.87 (Palgi *et al*., 2020*a*). In this study, Cronbach's *α* for peritraumatic distress was 0.84.

### Data analysis

First, Pearson correlations were conducted to establish preliminary associations between the study variables. Next, examination of the moderation model was conducted using the IBM SPSS statistic package (SPSS-25) to conduct a hierarchical regression analysis, where peritraumatic tress was regressed on the following variables across steps: Background characteristics were entered in the first step of the multiple hierarchical regression, COVID-19-related covariates (chronic diseases and exposure to COVID-19) in the second step, COVID-19 health worries and subjective accelerated aging in the third step, and finally the interaction between COVID-19 health worries and subjective accelerated aging was entered in the fourth step. The moderation model (Model 1) was tested, using the PROCESS 3.1 macro for SPSS (Hayes, 2017). A preliminary multicollinearity test was performed to confirm that the regression hypotheses were met. Results showed that the tolerance of all independent variables (age, gender, education, self-rated health, marital status, chronic diseases, health worries, and subjective accelerated aging) ranged from 0.654 to 0.970; the variance inflation factor ranged from 1.031 to 1.526, revealing no multicollinearity (O'Brien, 2007).

## Results

COVID-19 health worries were positively associated with subjective accelerated aging (*r* = 0.15, *p* < 0.05) and with peritraumatic distress (*r* = 0.40, *p* < 0.01). Subjective accelerated aging was positively associated with peritraumatic distress (*r* = 0.38, *p* < 0.01). It was important to control for these background characteristics as they were associated with at least one of the study's variables and there has been some variability regarding participants' exposure levels to various forms of COVID-19 stressors (e.g. isolation) (for further information, see Table 1).

In order to examine the study's hypotheses, a multiple hierarchical regression was conducted. The results showed that older adults who reported higher levels of COVID-19 health worries also reported higher levels of peritraumatic distress symptoms (*β* = 0.345, *t* = 5.879, *p* < 0.001). In addition, those who reported higher levels of subjective accelerated aging also reported higher levels of peritraumatic distress symptoms (*β* = 0.301, *t* = 5.134, *p* < 0.001). Finally, the interaction between COVID-19 health worries and subjective accelerated aging predicted peritraumatic distress (*B* = 2.186, *β* = 0.299, *t* = 5.298, *p* < 0.001). This interaction explained an additional 7.74% of the variance (see Table 2).

**Table 2.** Summary of the moderation model for variables predicting peritraumatic distress among older adult (*N* = 277)

	Δ*R*^2^	*B*	*β*	*t*	*p*
**Step 1**	0.092***				
Age		−0.14	−0.140	−2.11	0.036
Gender		1.59	0.108	1.56	0.119
Education		−1.58	−0.212	−3.18	0.002
Self-rated health		−1.03	−0.136	−2.08	0.039
Marital status		−0.53	−0.036	−0.53	0.596
**Step 2**	0.023				
Chronic diseases		−2.42	−0.179	−2.37	0.019
Exposure to COVID-19		−0.112	−0.018	−0.29	0.774
**Step 3**	0.212***				
Subjective accelerated aging		2.245	0.301	5.13	<0.001
COVID-19 health worries		2.530	0.345	5.88	<0.001
**Step 4**	0.77***				
COVID-19 health worries × subjective accelerated aging		2.186	0.299	5.30	<0.001

Total *R*^2^ = 0.404***.

*Note:* Gender (dummy): 1 – male, 2 – female.

*p < 0.05, **p < 0.01, ***p < 0.001.

**Fig. 1.**
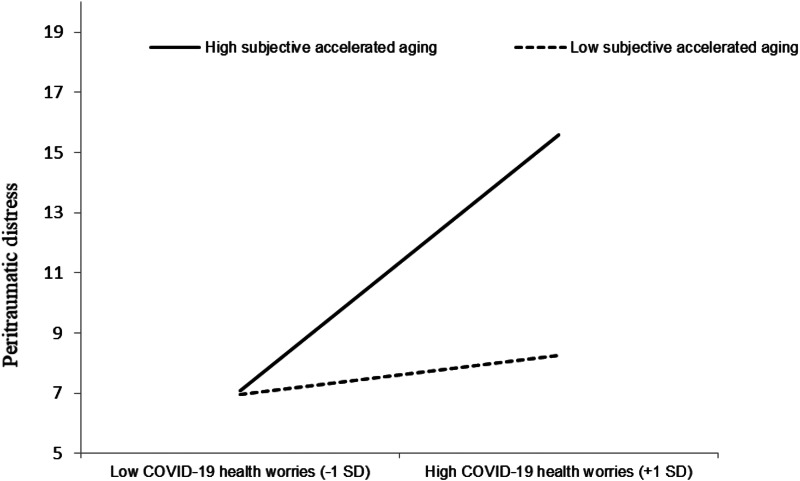
The moderating effect of subjective accelerated aging on the association between health worries and peritraumatic distress.

Probing this interaction, it was found that for older adults who reported a slow rate of aging (i.e. 1 s.d. below the mean; lower subjective accelerated aging), each additional point in COVID-19 health worries score was associated with a non-significant increase of 0.72 points in peritraumatic distress symptoms (*B* = 0.72, *t* = 1.35, *p* = 0.18). However, for older adults reporting a faster rate of aging (i.e. 1 s.d. above the mean; high subjective accelerated aging), each additional point in COVID-19 worries score was associated with a significant increase of 4.64 points in the level of peritraumatic distress symptoms (*B* = 4.64, *t* = 8.16, *p* < 0.001) (see Fig. 1).

## Discussion

To the best of our knowledge, the present study is the first to examine the relationship between COVID-19 health worries and peritraumatic distress among older adults, and in particular, the moderating role of subjective accelerated aging in this relationship. As hypothesized, older adults who reported higher levels of COVID-19 health worries also reported higher levels of peritraumatic distress symptoms. Moreover, those who felt they were aging fast (i.e. higher subjective accelerated aging levels) reported a higher level of peritraumatic distress symptoms. Finally, the interaction between these measures accentuates that the relationship between COVID-19 health worries and peritraumatic distress was stronger among people who reported the feeling they were aging faster. The significance of these findings will now be discussed in detail.

In line with the first hypothesis, high levels of COVID-19 health worries among older adults were found to be related to higher levels of peritraumatic distress. This association highlights the physical and psychological distress older adults experience during a pandemic. The present findings are in line with the association found between exposure to mass trauma events, such as natural catastrophes and adverse mental health outcomes, in particular PTSD (Makwana, 2019). In addition, the present findings are consistent with preliminary findings among adults during the pandemic that showed associations between higher levels of COVID-19 health worries and anxiety (Bergman *et al*., 2020) and between higher levels of COVID-19 health worries and death anxiety (Ring *et al*., 2020).

Consistent with our second hypothesis, older adults who felt they were aging faster also reported higher levels of peritraumatic distress. The findings underscore the effect of subjective accelerated aging when coping with stressful life events, such that older adults who sense their aging has accelerated are less resilient in the context of coping with COVID-19. The current findings align with the association found between subjective age and neurobiological accelerated aging (e.g. Tsur *et al*., 2018), as well as the association found between subjective accelerated aging and PTSD symptoms (Palgi, 2020). In addition, as noted by Palgi (2020), the concept of subjective accelerated aging appears to be an integrated part of the concept of subjective views of aging (Wurm *et al*., 2017) and these findings demonstrate its utility as an additional aspect of this concept.

In interpreting the interaction found in the present study between COVID-19 health worries and subjective accelerated aging, it can be speculated that older adults who felt they were aging faster were more vulnerable to the negative concomitants of COVID-19 worries, i.e. those who viewed themselves as rapidly progressing toward old age, an age at higher risk for severe COVID-19 illness, had no buffer against COVID-19 worries. Therefore, it seems that under the current context, where medical circumstances emphasized older ages as a risk factor for a severe illness, feeling one's aging process as slower is a potential resource that can render one more immune to the effects of health worries. It may be that the term accelerated aging is oversensitive to physical health worries due to the health risk involved. Moreover, peritraumatic distress was not associated with any vulnerability measures of the COVID-19 pandemic (i.e. chronological age, chronic disease, exposure to COVID-19 stressors), but rather only to the subjective measure of subjective accelerated aging. This finding coincides with previous studies that revealed that older chronological age is not associated with worse mental health outcomes during the COVID-19 pandemic (e.g. Pieh *et al*., 2020). In this light, the present study highlights the effect that subjective accelerating aging and excessive worrying may have on mental health outcomes in the older population during a pandemic (see Fig. 1).

The study has several limitations. First, it used a cross-sectional design; therefore, causality cannot be inferred from the current findings. Second, the study sample was based on an online design, and may be biased toward older adults with technological knowledge. Third, there was no measurement of pre-pandemic levels of health worries or levels of PTSD symptoms (the participants were asked to report their level of health worries and peritraumatic symptoms since the COVID-19 outbreak). Fourth, COVID-19 health worries were assessed by a scale that was previously published (Bergman *et al*., 2020; Ring *et al*., 2020; Grossman *et al*., 2021), nevertheless this measure is not standardized. Future studies should use standardized measures (e.g. Ahorsu *et al*., 2020*a*, *b*; Bitan *et al*., 2020; Lee, 2020; Taylor *et al*., 2020). Finally, similar to other views of aging (e.g. distance to death, subjective age), accelerated aging is also a single item. Future studies may focus on unpacking this concept into further potential domains.

## Conclusions

The present study is the first to examine the concept of subjective accelerated aging and its relationship with peritraumatic distress during the COVID-19 pandemic among older adults. The findings have theoretical and practical implications for the prevention of peritraumatic distress during the COVID-19 pandemic, as well as other natural disasters.

Theoretically, the findings rejoin earlier results reviewed above and further demonstrate that similar to the biological process of accelerated aging, there appears to be a psychological process by which individuals assess the rate of their aging, which seems to be linked with psychiatric symptoms. We recommend future studies to examine the directionality of this relationship and its contribution to mental health. We also recommend that future prospective studies investigate the mechanisms connecting subjective and objective measures of accelerated aging.

On a practical level, the rise in health worries since the COVID-19 outbreak is understandable (Kobayashi *et al*., 2020); therefore, the findings in the current study offer preliminary support for the potential relevance of suitable interventions during the COVID-19 pandemic that are aimed at reducing health worries. In particular, the findings underscore the importance of identifying health worries among older adults, and more so among those who experience subjective accelerated aging in the face of the COVID-19 pandemic. The combination of health worries together with subjective accelerated aging pinpoints a group at higher risk for developing increased levels of peritraumatic distress symptoms. Preventative interventions for adverse psychological effects can be implemented among this group. Finally, another potential approach is to focus on the subjective feeling of accelerated aging; the development of suitable interventions for promoting the feeling of aging in a less accelerated fashion should be promising for older adults during stressful life events.
